# Respiratory Morbidity of Roadside Shopkeepers Exposed to Traffic-related Air Pollution in Bhopal, India

**DOI:** 10.5696/2156-9614-9.21.190305

**Published:** 2019-03-07

**Authors:** Sajal De, Gagan Deep Singh Kushwah, Dharmendra Dharwey, Devika Shanmugasundaram

**Affiliations:** 1 Department of Pulmonary Medicine, National Institute for Research in Environmental Health, Kamla Nehru Hospital Building, Gandhi Medical College Campus, Bhopal, India; 2 Department of Epidemiology, National Institute for Research in Environmental Health, Kamla Nehru Hospital Building, Gandhi Medical College Campus, Bhopal, India

**Keywords:** respiratory morbidity, traffic related-air pollution, shopkeeper

## Abstract

**Background.:**

Traffic-related air pollution (TRAP) is a major source of ambient air pollution in urban areas. Shopkeepers of heavily trafficked roadside shops are persistently exposed to high levels of TRAP.

**Objectives.:**

To estimate the prevalence of respiratory morbidity in shopkeepers of shops in heavily trafficked roadside areas in Bhopal city (India) and to determine any association with long term exposure to TRAP.

**Methods.:**

This cross-sectional study was conducted among 251 shopkeepers working in roadside shops of three major traffic corridors in Bhopal city. The demographic profile and prevalence of respiratory morbidity were collected by administering a validated questionnaire. The total exposure period (TEP) to TRAP was calculated for each individual by multiplying their work duration (in years) and average working hours per day. Odds ratios were calculated to estimate the association of TEP with respiratory morbidity.

**Results.:**

The age of the study population was 44.8±13.5 years old and 95% were male. Nearly 55% of the shopkeepers reported at least one respiratory symptom. The prevalence of bronchial asthma, chronic bronchitis, breathlessness, and cough was 3.6% (95% confidence interval (CI): 1.9–6.7), 13.9% (95% CI: 10.2–18.8), 41.8% (95% CI: 35.9–48.0), and 18.3% (95% CI: 14.0–23.6), respectively. The adjusted risk ratios of bronchial asthma 2.17 (95% CI: 0.35–13.41), chronic bronchitis 1.42 (95% CI: 0.58–3.48), breathlessness 1.71 (95% CI: 0.94–3.11), and cough 0.97 (95% CI: 0.47–2.03) for those with a TEP over 100.

**Conclusions.:**

Shopkeepers working in heavily trafficked roadside shops suffer from respiratory morbidity and the risk increases with higher TEP. Total exposure period is a valuable indicator to estimate the effects of long-term TRAP exposure.

**Informed Consent.:**

Obtained

**Ethics Approval.:**

The study was approved by the Institutional Ethics Committee of the National Institute for Research in Environmental Health (Bhopal, India).

**Competing Interests.:**

The authors declare no competing financial interests.

## Introduction

Outdoor air pollution accounted for 6% of the total disease burden in India in 2016.^1^ Traffic-related air pollution (TRAP) is the main source of ambient air pollution in the majority of Indian cities. Motor vehicle exhaust emits diverse pollutants, including particulate matter (PM), lead, carbon monoxide, sulfur dioxide, oxides of nitrogen, unburned fuel, partly oxidized hydrocarbons, benzene, and polycyclic aromatic hydrocarbons.^2^ Particulate matter and other components of vehicular exhaust penetrate the lung and initiate inflammation, a key step towards developing TRAP-induced adverse health effects.^3^

The adverse health effects of TRAP are usually greater than industrial pollutants, as TRAP is released near ground level and remains there for longer periods, especially where roads are enclosed by high-rise buildings. Epidemiological studies have established the adverse health effects of TRAP exposure and its association with higher cardiorespiratory morbidity and mortality.^4^ Individuals working or residing adjacent to roads with heavy traffic are persistently exposed to high levels of TRAP. The literature on the effects of chronic TRAP exposure on respiratory morbidity among roadside shopkeepers in India is sparse.^5^,^6^ Bhopal city is the capital of Madhya Pradesh state and is situated in central India. Bhopal has a subtropical climate; hot summers, cool and dry winters, and a humid monsoon season. Due to rapid urbanization, traffic volume in Bhopal city has increased over the last two decades and TRAP is major source of ambient air pollution in the city.^7^

The aim of present study was to estimate the prevalence of respiratory morbidity in shopkeepers working in heavily trafficked roadside shops in Bhopal city and to determine its association with duration of work, a surrogate for long-term exposure to TRAP.

## Methods

**Figure 1:**
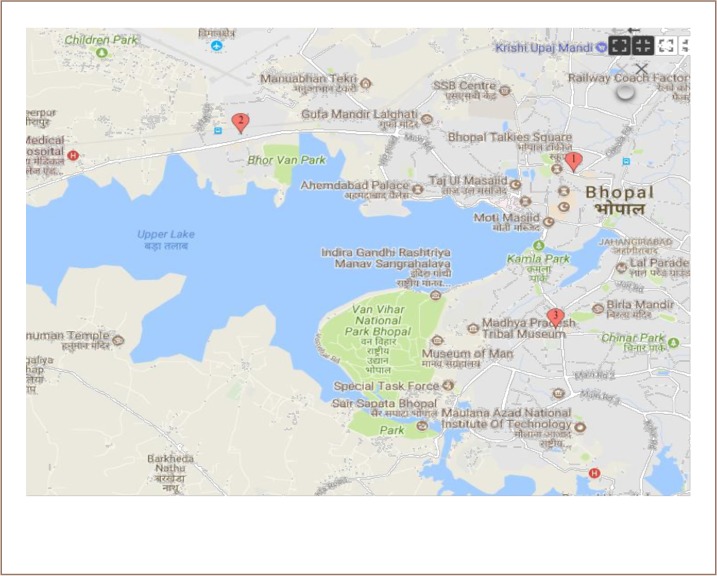
Map of Bhopal city showing the areas from where the shopkeepers were recruited. 1) Area I: along Hamidia Road; 2) Area II: along Bairagarh Road; and 3) Area III: New Market. Source: https://www.google.co.in/maps

A cross-sectional descriptive study was carried out in Bhopal. The study was approved by the Institutional Ethics Committee of the National Institute for Research in Environmental Health (Bhopal, India). Bhopal city is divided into two distinct parts: the old and new city. The roads of the old city are narrow, congested and have a high volume of heterogeneous traffic. Remote sensing and geographic information system-based mapping demonstrate that major traffic corridors of Bhopal city are responsible for poor ambient air quality.^8^ In the present study, we included road-facing shops located on either side of three major traffic corridors of old Bhopal City (*Figure 1*). Area I was located around Hamidia Road, a well-known congested and busy road of old Bhopal city. The intercity bus terminal and rail station are situated on this road. The vehicles on Hamidia Road include passenger cars and heavy vehicles and this area has an estimated traffic volume of 72,000 passenger car unit (PCU).^9^ Area II was located at Bairagarh Road, a part of the national highway connecting Bhopal city with Indore, another major city in Madhya Pradesh. Vehicle traffic on this road is mainly comprised of heavy vehicles and the estimated traffic volume is 19,000 PCU.^9^ Area III was New Market, a commercial hub of Bhopal city. This market is encircled by major traffic corridors and the shops are located within 100 m from either roads. The vehicles on this corridor are mostly comprised of passenger cars and the estimated traffic volume is 51,000 PCU.^9^ Shops in area I mostly deal with spare parts of machinery and electrical goods, whereas shops in areas II and III are primarily involved in the garment business.

**Abbreviations**
*CI*: Confidence interval
*CPCB*: Central Pollution Control Board
*INSEARCH*: Indian Study on the Epidemiology of Asthma, Respiratory Symptoms and Chronic Bronchitis
*IQR*: Interquartile range
*PCU*: Passenger car unit
*PM*: Particulate matter
*TEP*: Total exposure period
*TRAP*: Traffic-related air pollution
*WHO*: World Health Organization

### Study population

Shopkeepers from the three study areas were approached to participate in the present study. We enrolled only shop owners who were actively involved in the management of their shops, to ensure that this would reflect actual exposure. Other shop employees were excluded as they often change jobs, thus their actual exposure to TRAP could not be determined. Restaurant owners were also excluded as they might have additional exposure to kitchen fumes. The nature of the study was explained and verbal consent was obtained from each participant prior to data collection.

### Data collection

Information on various respiratory symptoms was collected using a pre-validated Hindi translation of the Indian Study on the Epidemiology of Asthma, Respiratory Symptoms and Chronic Bronchitis (INSEARCH) questionnaire.^10^ The diagnosis of bronchial asthma and chronic bronchitis was established as instructed in the INSEARCH questionnaire. The diagnosis of bronchial asthma was established by affirmative responses to a history of wheezing or tightness of the chest, plus one of the following: a history of previous diagnosis of asthma, an attack of asthma and/or use of medication for asthma in the past 12 months. Similarly, the diagnosis of chronic bronchitis was based on presence of cough with expectoration for > 3 months and at least one of the following: cough first thing in the morning and/or ejecting phlegm from the chest in the morning. Breathlessness was defined as an affirmative response to at least one of the following four criteria: breathlessness in the morning, breathlessness on exertion, breathlessness without exertion, and/or breathlessness at night. Cough was defined as the report of cough either in the morning or at night.

### Sample size calculation

A previous study from Bangalore (India) reported a 12.4% and 32.2% prevalence of cough and breathlessness in roadside shopkeepers, respectively.^5^ Based on the above observations, we expected that the prevalence of either cough or breathlessness in our study population would be 20%.

With a sample size of 251 subjects and a margin of error set at 5%, we used the formula n = Z^2^^*^ P ^*^ (1 - P) / d^2^, where Z was the level of confidence (95%), P was the expected prevalence (20%), and d was the allowable error (5%).

### Statistical analysis

All baseline variables and outcomes were summarized as percentages, median with interquartile range (IQR), and mean ±standard deviation. The prevalence of respiratory symptoms with 95% confidence interval (CI) was calculated based on the Wilson score method. The association of respiratory symptoms with location was assessed using the chi-square test and Fisher's exact test based on the data distribution. To standardize the duration of TRAP exposure, we calculated the total exposure period (TEP) of each individual by multiplying their work duration (in years) and working hours per day. Longer periods of exposure to TRAP are necessary to perceive health impacts. Thus, TEP measurements were categorized into three categories (TEP ≤100, TEP >100 - ≤250, and TEP>250) based on the TEP distribution of the study population to examine the prevalence of respiratory morbidity in each TEP measurement. Univariate logistic regression was performed to identify the association of TEP, smoking, and age with different respiratory morbidities (i.e. breathlessness, cough, bronchial asthma, and chronic bronchitis). Multivariate logistic regression analysis to estimate the effect of TEP on respiratory morbidity was carried out using TEP ≤ 100 (i.e. worked for 10 hours per day for 10 years) as a reference. The odds ratios with 95% CI were presented. Stata 13.1 software for windows (Stata Corp, College Station, TX, USA) was used for the statistical analysis. For all analyses, a two-tailed P value <0.05 was considered to be statistically significant.

## Results

The present study was carried out between September 2017 and January 2018. A total of 485 shopkeepers were approached for recruitment and 251 (52%) participated. The main reason shopkeepers gave for refusing to participate was that they were preoccupied with customers. The number of shopkeepers recruited from area I, II, and III were 85 (33.9%), 68 (27.1%) and 98 (39%), respectively. All participants resided in houses that were at different locations from their shops. The demographic characteristics of the study population did not differ between study areas. The education level of study participants was as follows: 34% had a high school graduate level or higher, 50% had a middle to high school education, and 16% had a primary school level education or lower. The majority of the study population was male (97%) and their mean age was 44.8±13.5 years old. The mean working hours per day was 10.4±2.0 and median work duration was 16 years (IQR: 10–26). The median TEP of the study population was 165 (IQR: 84–300). Smoking history longer than one year was reported by 15.5% of study participants. The prevalence of previously diagnosed diabetes and hypertension among the participants was 10% and 17.9%, respectively.

The prevalence of different respiratory symptoms in the three different areas is summarized in Table 1. With a few exceptions, the prevalence of respiratory symptoms was comparable in all three areas. Nearly 55% of the shopkeepers reported at least one respiratory symptom. Among all respiratory symptoms, exertional breathlessness was the most common symptom and was reported by 35.5% (95% CI: 29.8–41.6) of shopkeepers. The prevalence of bronchial asthma and chronic bronchitis was 3.6% (95% CI: 1.9–6.7) and 13.9% (95% CI: 10.2–18.8), respectively. The prevalence of both cough and breathlessness was 18.3% (95% CI: 14.0–23.6) and 41.8% (95% CI: 35.9–48.0), respectively.

**Table 1:** The Prevalence of Respiratory Symptoms in Shopkeepers as Reported- on the INSEARCH Questionnaire

**Variables**	**Area-I**	**Area-II**	**Area-III**	**Total**	
	**(N=85)**	**(N=68)**	**(N=98)**	**(N=251)**	
	n (%)	n (%)	n (%)	%	95% CI^#^
Wheeze	14(16.5)	5 (7.4)	12 (12.2)	12.4	(8.8, 17.0)
Morning breathlessness	26 (30.6)^*^	12 (17.6)	13 (13.3)	20.3	(15.8, 25.7)
Breathlessness on exertion	32 (37.6)	21 (30.9)	36 (36.7)	35.5	(29.8, 41.6)
Breathlessness without exertion	19 (22.4)^*^	2 (2.9)	4(4.1)	10.0	(6.8, 14.3)
Breathlessness at night	15 (17.6)	6 (8.8)	15 (15.3)	14.3	(10.5, 19.2)
Cough at night	17 (20.0)	7 (10.3)	13 (13.3)	14.7	(10.9, 19.7)
Cough in morning	14 (16.5)^*^	3 (4.4)	8 (8.2)	10.0	(6.8, 14.3)
Phlegm in morning	19 (22.4)	10 (14.7)	15 (15.3)	17.5	(13.3, 22.7)
Breathing never satisfactory	4 (4.7)	2 (2.9)	6 (6.1)	4.8	(2.8, 8.2)^^^
Usually breathless	24 (28.2)^*^	15 (22.1)	12 (12.2)	20.3	(15.8, 25.7)
Chest tightness during dust exposure	16 (18.8)	9 (13.2)	29 (29.6)^*^	21.5	(16.9, 27.0)
Breathlessness during dust exposure	20 (23.5)	11 (16.2)	30 (30.6)	24.3	(19.4, 30.0)
Any of the above symptoms	52 (61.2)	31 (45.6)	54 (55.1)	54.6	(48.4, 60.6)
Bronchial asthma	3 (3.5)	3 (4.4)	3 (3.1)	3.6	(1.9, 6.7)
Chronic bronchitis	14 (16.5)	9 (13.2)	12 (12.2)	13.9	(10.2, 18.8)

^#^ Wilson score confidence interval;

^^^ Fisher's exact test;

^*^ p <0.05.

**Figure 2:**
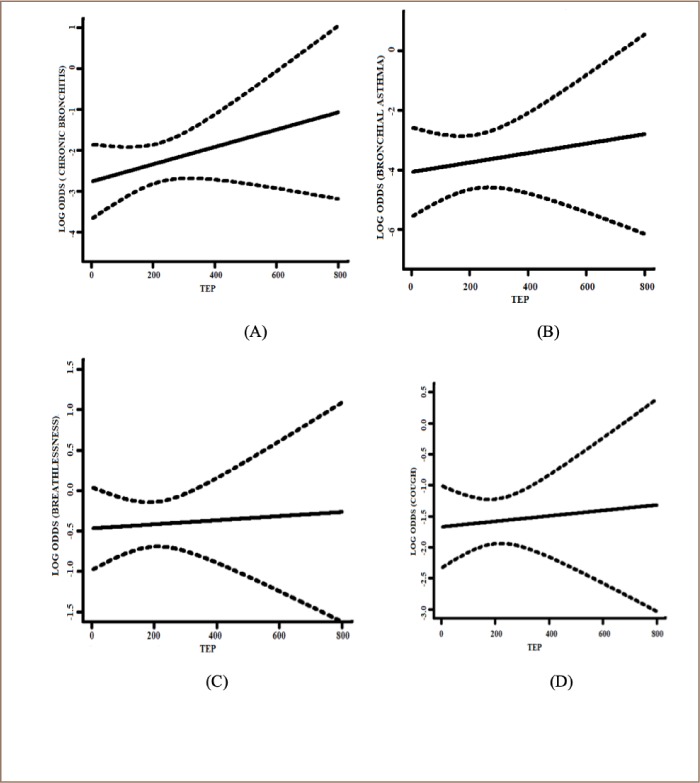
Plots of total exposure period (TEP) and adjusted log of the odds ratio for different respiratory symptoms with confidence bands (dotted lines). A: Association of TEP with chronic bronchitis. B: Association of TEP with bronchial asthma. C: Association of TEP with breathlessness. D: Association of TEP with cough

The prevalence of respiratory morbidity increased with higher TEP and the highest risk was observed for breathlessness (*Table 2*). The association of different respiratory symptoms with TEP, age, and smoking are presented in Table 3. Smoking was strongly associated with higher respiratory morbidity, especially for chronic bronchitis and bronchial asthma. After adjusting for age and smoking status, the log odds for having respiratory morbidity showed increasing trends with longer TEP (*Figure 2*). Multivariate analysis showed an adjusted odds ratio of chronic bronchitis 1.42 (95% CI: 0.58–3.48), bronchial asthma 2.17 (95% CI: 0.35–13.41), breathlessness 1.71 (95% CI: 0.94–3.11), and cough 0.97 (95% CI: 0.47–2.03), respectively, for those with a TEP greater than 100.

**Table 2:** Prevalence of Respiratory Symptoms Stratified by Total Exposure Period

**Respiratory symptoms**	TEP ≤100 (n=78) Prevalence (95% CI)^*^	TEP >100-≤250 (n=103) Prevalence (95% CI)^*^	TEP >250 (n=70) Prevalence (95% CI)^*^
Breathlessness	33.33 (23.87, 44.36)	48.54 (39.11, 58.07)	41.43 (30.63, 53.12)
Cough	17.95 (11.00, 27.90)	17.48 (11.35, 25.94)	20.00 (12.30, 30.82)
Bronchial Asthma	1.28 (0.23, 6.91)	3.88(1.52, 9.56)	5.71 (2.24, 13.79)
Chronic Bronchitis	11.54 (6.19, 20.50)	13.59 (8.27, 21.53)	17.14 (10.09, 27.62)

^*^Wilson score-based confidence interval

Abbreviation: TEP, total exposure period

**Table 3:** Univariate Analysis to Assess the Association of Respiratory Morbidity with Total Exposure Period (TEP), Age and Smoking

	**Odds ratio (95% Confidence interval)**		
**Respiratory symptoms**	**TEP^$^**	Age^*^	**Smoking**
Breathlessness	1.04 (0.86, 1.25)	1.03 (0.86, 1.24)	1.78 (0.89, 3.53)
Cough	1.05 (0.83, 1.32)	1.03 (0.81, 1.30)	1.69 (0.76, 3.76)
Bronchial Asthma	1.25 (0.81, 1.93)	1.24 (0.76, 2.04)	4.73 (1.21, 18.48)
Chronic Bronchitis	1.10 (0.86, 1.42)	0.93 (0.71, 1.22)	7.07 (3.20, 15.62)

^$^ Unit of increase 100 units (10 years of 10 hours per day of work);

^*^ Unit of increase 10 years

Abbreviation: TEP, total exposure period

## Discussion

The present study assessed the prevalence of respiratory morbidity in roadside shop owners exposed to TRAP. The results highlight the high prevalence of respiratory morbidity among study participants and longterm exposure to TRAP was shown to be a risk factor.

Vehicular exhaust produces small-scale spatial variations in air pollution and affects the background air pollution level. The vehicular pollutant level gradually decreases with distance from roads and thus distance from roads is an important factor influencing the level of TRAP exposure.^11^ Local climatic conditions, composition of traffic on the road (i.e. two- or three-wheeled vehicles, passenger cars, commercial vehicles, etc.), vehicle speed (use of brakes and accelerator), quality of fuel, average age of vehicles, etc. effect pollutant levels. Traffic congestion due to narrow and congested roads leads to lower vehicle speed, frequent slowdowns and accelerations and thus increases vehicle emissions and degrades ambient air quality.^12^ Weak vehicular exhaust emission regulations, poor compliance to existing emission standards, poor fuel quality, and a large proportion of older vehicles play a crucial role in producing poor air quality in older highly populated cities in developing countries. Traffic-related air pollution is worse during winter months when temperature inversion occurs. A street canyon effect is formed in urban streets where adjoining high-rises and densely packed buildings prevent the dispersion of TRAP via changing wind velocities and directions. The presence of street canyons further intensifies ambient pollution and can potentiate the adverse health effects of TRAP.^13^ All shops in the present study were situated on the ground floor with open entrances towards the road, thus shopkeepers directly get exposure to TRAP. The majority of the shops (98%) were not air conditioned. Previous studies have shown that airconditioning in roadside shops can inadequately protect workers from the respiratory effects of TRAP.^14^

**Figure 3:**
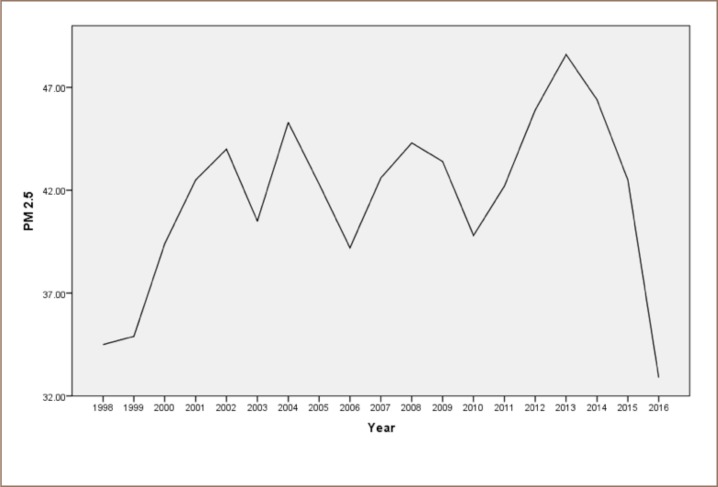
Concentration of ambient PM2.5 in Bhopal city from 1998 to 2016^5^,^18^

In India, real-time ambient air pollution data are available for a few cities under the National Ambient Monitoring Program, operated by the Central Pollution Control Board (CPCB, New Delhi, India). Prior to 2018, air pollution in Bhopal was monitored by manual stations operated by the Madhya Pradesh Pollution Control Board. From 2011 to 2016, the average concentrations of respirable suspended PM (PM of aerodynamic diameter <10 μm) in Bhopal City ranged from 101.0 to 354.6 μg/m^3^ (the prescribed annual limit set by the World Health Organization (WHO) and the CPCB standard are 20 μg/m^3^ and 60 μg/m^3^, respectively), the average SO_2_ ranged from 2 to 5 μg/m^3^ (the prescribed annual limit per the WHO and CPCB standards are 20 μg/m^3^ and 50 μg/m^3^, respectively), and average nitrogen oxides ranged from 9.6 to 29.9 μg/m^3^ (the prescribed annual limit per the WHO and CPCB is 40 μg/m^3^).^15–17^ From 1990 to 1995, respirable suspended PM in Bhopal ranged from 207 to 280 μg/m^3^, SO_2_ ranged from 8.5 to 16.5 μg/m^3^, and nitrogen oxides ranged from 7.8 to 25.2 μg/m^3^.^7^ The concentration of PM_2.5_ and other important air pollutants known to have adverse health effects such as black carbon, fine and ultrafine particles, and volatile organic compounds have never been monitored. Based on satellite data, the average annual concentration of PM_2.5_ in Bhopal city during 1998 to 2014 was 49.9 ± 6.7 μg/m^3^, above the prescribed upper limits of the WHO (10 μg/m^3^) and CPCB (40 μg/m^3^) standards (*Figure 3*).^16–18^

The risk of cardiopulmonary mortality is higher for those residing near major roads and experiencing long-term exposure to PMs from TRAP.^19^ Increased prevalence of respiratory symptoms and an excess FEV_1_ decline have been observed in adult females chronically exposed to higher levels of PMs from automobile exhaust.^20^ The severity of the health effects of TRAP vary due to different concentrations and compositions of pollutants, along with the genetic susceptibility of the exposed population.^21^ Noomnual et al. examined thirty young Thai street vendors exposed to TRAP and observed a 27%, 13%, 3%, 10% and 7% prevalence of cough, phlegm, wheeze, chest tightness, and shortness of breath, respectively.^22^ Kongtip et al. examined the health effects of TRAP exposure in 77 street vendors from Thailand and reported a 7.8%, 22.4%, and 0.59% prevalence of cough, phlegm, and chest tightness, respectively.^23^

Ramesh et al. evaluated 121 shopkeepers in India working for more than a year in shops located within 100 m radius of the national highway of Bangalore and observed a prevalence of cough, breathlessness, and asthma of 32.2%, 31.4%, and 12.4%, respectively.^5^ The respiratory morbidity of their study population was significantly higher than that of the general population of Bangalore City. Prakash et al. observed reduced lung function in roadside vendors in Punjagutta (Andhra Pradesh, India).^6^ Compared to previous studies, the present study had a larger sample size and demonstrated lower prevalence of cough (18.3%) and higher prevalence of breathlessness (41.8%). The present study was the first to use TEP to explore the association of respiratory morbidity with long term TRAP exposure. Gupta et al. observed traffic police personnel with > 8 years exposure to TRAP had lower lung function as compared with those with <8 years exposure.^24^ A limitation of the present study was that lung function was not evaluated and there was a lack of female shopkeepers and hence female study subjects.

The INSEARCH questionnaire used in the present study was developed to assess the overall burden of chronic respiratory diseases in India. The original INSEARCH study reported the prevalence of asthma and chronic bronchitis in adults as 2.05% and 3.49% respectively.^10^

In the present study, the prevalence of all respiratory symptoms, including bronchial asthma and chronic bronchitis in shopkeepers was higher compared to the INSEARCH study.

## Conclusions

The present study demonstrated that shopkeepers working in congested and heavily trafficked roadside shops suffer from respiratory morbidity and the risks are higher for those with longer TRAP exposure. In addition, it showed that TEP can be a valuable indicator to assess the cumulative effects of TRAP exposure. There is a need to increase the awareness of individuals working or living near heavily trafficked roads of the adverse health effects of TRAP exposure and to promote measures such as glass doors on road-facing fronts to reduce direct exposure. Finally, measures to reduce traffic congestion, widen urban roads, promote the use of cleaner fuels, and prohibition of polluting vehicles can help to reduce ambient TRAP.
